# The Frontal Fibrosing Alopecia Treatment Dilemma

**DOI:** 10.3390/jcm13072137

**Published:** 2024-04-07

**Authors:** Wiktoria Julia Krzesłowska, Anna Woźniacka

**Affiliations:** Department of Dermatology and Venereology, Medical University of Lodz, Hallera 1, 90-647 Lodz, Poland; wiktoria.julia.krzeslowska@gmail.com

**Keywords:** FFA, frontal fibrosing alopecia, FFA treatment, frontal hairline recession, inflammatory scalp disease, cicatricial alopecia, FFA management

## Abstract

Frontal fibrosing alopecia (FFA) is a type of cicatricial alopecia predominantly observed in postmenopausal women, with the incidence rising since its initial description in 1994. The exact etiopathogenesis of the disease has not been completely elucidated. FFA is characterized by an inflammatory process affecting the hair follicles of the fronto-temporal hairline, leading to its gradual recession. Eyebrows, particularly the lateral parts, may also be affected. Early diagnosis and an implementation of effective therapy to limit the inflammatory process are crucial in halting disease progression. Various treatment possibilities have been reported, including anti-inflammatory and immunosuppressive agents, as well as 5-alpha-reductase inhibitors, retinoids, and antimalarial agents. The use of phototherapy and surgical procedures has also been described. However, most available data have been obtained retrospectively, frequently consisting of descriptions of case reports or small case series, and not from randomized controlled trials. In addition, the etiopathogenesis of FFA remains unclear and its course unpredictable, occasionally being linked with spontaneous stabilization. Hence, no precise guidelines exist regarding treatment modalities. Therefore, the aims of this study were to provide a comprehensive review of the efficacy of existing therapeutic modalities for FFA and to highlight novel therapeutic options.

## 1. Aim and Methods

The aims of this study were to conduct a comprehensive review of the current state of knowledge regarding the treatment of FFA and to identify potential areas for further evaluation. To achieve these aims, an exhaustive search of references related to FFA published in PubMed was undertaken, using the search term “frontal fibrosing alopecia”. Articles with larger patient cohorts and reviews were included, along with publications featuring smaller patient samples that offer novel treatment approaches for FFA.

## 2. Introduction

Frontal fibrosing alopecia, first described in 1994 by Kossard et al., is a form of cicatricial alopecia [1,2,3]. While initially classified as a variant of lichen planopilaris (LPP), some consider it a distinct entity, mainly due to differences in clinical presentation [4,5]. Although global data on the prevalence of FFA are limited, its occurrence in the general population is estimated to be 0.015% [6]. The vast majority of FFA patients (97%) are women [4], mostly in the postmenopausal period (mean age of onset: 56–63 years) [4,6,7]; however, its frequency may be underestimated in men, due to a potential overlap with androgenetic alopecia. In addition, the mean age of onset in men (47 years) is lower than in women [4,6,8,9].

Although the etiopathogenesis of FFA remains unclear [10], it is believed that autoimmune, hormonal, environmental, and genetic factors may play a role [4,10,11,12]. It may also coexist with allergic diseases, rosacea, thyroid diseases, or tumors. It has been proposed that hormonal changes during pregnancy, breastfeeding, hysterectomy, and hormone or raloxifene therapy may also play a role, as well as exposure to sunscreen components, moisturizers, or sunlight [10,13,14,15,16,17,18,19].

## 3. Clinical Features and Diagnosis

FFA is characterized by a gradual, usually symmetrical regression of the hairline in the frontal or fronto-temporal region [12,13,17,20]. In 10–25% of cases, the occipital hairline is also affected [4,21]. At the sites of the above-mentioned hairlines, single stems of terminal hair may be observed (the “lonely hair” sign) [22,23]. Total or partial loss of eyebrows occurs in 64–94% of cases, with the lateral parts being particularly affected. In 25% of cases, hair loss also occurs on the axillae, groin, or extremities [7,24,25,26].

Some patients, mainly men and premenopausal women, present facial papules (FP). These non-inflammatory, monomorphic lesions indicate involvement of the vellus follicular units and are similar to the follicular red dots of the glabella (FRD—follicular red dots) also observed in FFA patients [4,27,28,29]. Additional facial manifestations include erythema, pigmented patches, and frontal vein prominence or depression [27]. Inflammation-related symptoms including pruritus, burning sensation, or trichodynia are also reported [4].

**Figure 1:**
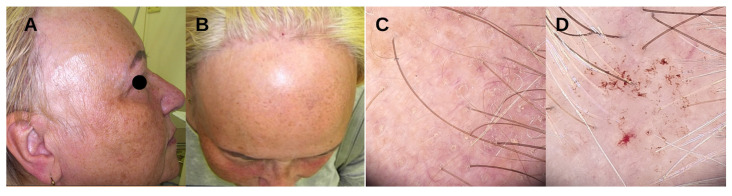
FFA; regression of fronto-temporal lines, pale skin in contrast with normal, photoaged skin, lateral eyebrow loss (**A**,**B**). Perifollicular hyperkeratosis, perifollicular erythema, and a lack of follicular units (**C**). Extravasated hemorrhages due to reduced erythema and perifollicular hyperkeratosis as a result of long-term topical corticosteroid use (**D**).

Trichoscopy reveals perifollicular erythema, a marker of FFA activity, as well as perifollicular hyperkeratosis and areas with a lack of follicular units [30]. Mild cases often present yellow dots, corresponding to the follicles having regrowth potential, and lack vellus follicular units. Advanced stages are characterized by black dots, broken or twisted hairs, branched blood vessels, and white patches [17]. Long-term use of topical corticosteroids may lead to the appearance of arborizing blood vessels and extravasated hemorrhages [31] (Figure 1). Eyebrow dermatoscopy reveals a “road sign image”, with hairs growing in different directions, together with black, yellow, and gray dots, diffuse erythema, and loss of follicular units. Perifollicular erythema and hyperkeratosis are usually not present [32]. Histological examination reveals abundant peri- and infundibular lymphocytic infiltration, keratinization in the infundibulum and hair follicle orifices, as well as apoptosis and vacuolar degeneration of basal layer keratinocytes. Scalp biopsy remains the gold standard for diagnosing FFA in equivocal cases, including men and premenopausal women [33,34]. However, diagnosis is mostly based on clinical examination. The diagnostic criteria proposed by Tolkachjov and Vañó-Galván et al. (Table 1) may aid in identifying the condition [35,36].

## 4. Disease Severity

### 4.1. Five-Point Severity Scale

The severity of FFA can be assessed using a five-point scale proposed by Vañó-Galván et al. (Table 2). The cicatricial area largest measure is defined as the largest measure [cm] of cicatricial skin produced by the recession of the frontal and temporal hairline [4]. Grades I and II indicate mild cases, and grades III, IV, and V severe FFA [4,6].

Additionally, the International FFA Cooperative Group (IFFACG) have proposed the following principles to provide a standardized approach for evaluating hairline recession. The degree of frontal hairline recession is determined by the distance from the superior edge of forehead wrinkling to the area of maximum hair density in the central frontal scalp, recorded in centimeters with one decimal point precision. For the temporal recession, the same procedure is applied bilaterally, measuring the distance from the superior edge of forehead wrinkling to the area of maximum hair density in each temporal area. For those having had previous botulinum injections, the end of photodamaged skin substitutes for the superior edge of forehead wrinkling [37].

### 4.2. FFASI and FASS

Disease assessment can also be performed using the Frontal Fibrosing Alopecia Severity Index (FFASI) and the Frontal Fibrosing Alopecia Severity Scale (FFASS). However, both have limitations, and their use remains controversial.

In addition to the features typical of FFA, the FFASI also takes into account characteristics such as nail, oral mucosa, or genital involvement. They are typical for lichen planus, which some authors consider a distinct entity from FFA. Moreover, this scale is complex, encompassing two sections, thereby demanding considerable time for routine clinical implementation [38].

The FFASS (Table 3) lacks trichoscopic evaluation and was developed based solely on data from one center, with patients exclusively being Caucasian women. Some authors have indicated that due to the potential linkage of FFA with LPP or traction alopecia among African and Asian individuals, the use of the FFASS in these populations may confuse the clinical assessment and potentially overestimate FFA [39,40].

As a result of these limitations, these scales are not typically applicable in selecting the appropriate form of the treatment and are not widely used in clinical practice; nevertheless, the FASS may be utilized for evaluating the efficacy of the treatment and may serve as an indicator for clinicians while considering treatment modifications.

### 4.3. Frontal Fibrosing Alopecia Global Staging Score and ALODEX-FFA

In response to the limitations posed by the previous scales, the IFFACG proposed the Frontal Fibrosing Alopecia Global Staging Score (FFAGSS) implemented in Table 4, as well as the Alopecia Density and Extent Score for FFA (ALODEX-FFA). The FFAGSS includes an assessment of the five common FFA characteristics: scalp hair loss (S), eyebrow loss (E), facial papules (P), prominent forehead veins (V), and hyperpigmentation (H). As such, the staging for a particular patient is as follows: S (0–4), E (0–2), P (0–1), V (0–1), H (0–1).

The ALODEX-FFA is determined by summing the hair density loss, scored from 1 to 5 (0 = 0–49%, 1 = 50–74%, 2 = 75–89%, 3 = 90–99%, 4 = 100%), in each of the 1% areas and dividing by 100. Additionally, the authors also propose assessing the severity of perifollicular hyperkeratosis and perifollicular erythema using the SALT 3 criterion: 0 = none, 1 = mild, 3 = severe [37].

## 5. Clinical Course and Prognostic Factors

**Figure 2:**
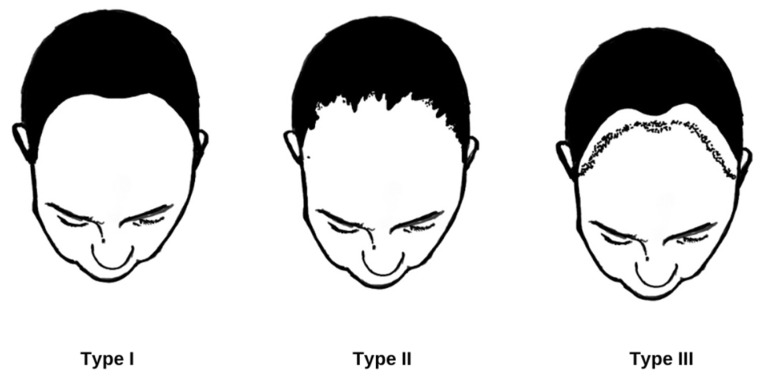
Clinical patterns of FFA presentation. Type I, “linear pattern”: a band of uniform frontal hairline recession. Type II, “diffuse pattern”: a diffuse or zigzag band-like alopecia of the frontal hairline with loss of hair density behind the hairline. Type III, “pseudo fringe-sign pattern” (original authors’ sketch).

FFA is a chronic disorder with an unpredictable course [41]. It can begin and progress insidiously, remain stable for a long time, progress rapidly, or spontaneously stabilize after several years of progression [4,5,7]. Without treatment, gradual hairline regression at a rate of 0.2 to 2.1 cm per year is observed [4]. It is believed that the hair loss resulting from FFA is irreversible [40]; however, hair regrowth has been reported in affected areas in some cases [4,42]. Older age at onset [42], higher BMI [13], peripheral hair loss, eyelash loss, FP [4], or coexistence of rosacea are associated with a severe disease course [43], while younger age at onset [26] corresponds to a milder course. Regarding the clinical types of FFA (Figure 2), the best prognosis is associated with Type III and the worst with Type II. Type I, the most common, has an intermediate prognosis [41]. Additionally, the presence of follicular fluorescence on ultraviolet-enhanced trichoscopy (UVET), known as the “starry sky sign”, may indicate a potential for hair regrowth [44].

## 6. Treatment

The main aims of treatment are to ameliorate symptoms and to suppress disease progression [7,24,45]. A positive response is manifested as stabilization, i.e., cessation of hairline regression, or improvement, i.e., hair regrowth; a negative response is considered as progressive hairline recession [4]. Some authors define disease stabilization as the absence of inflammation on biopsy; however, biopsy is an invasive procedure and is not routinely performed [46,47].

FFA treatment commonly consists of combined therapy, involving topical and systemic treatments [45,48]. Selected therapeutic options are presented in Table 5.

### 6.1. Local Treatment

#### 6.1.1. Anti-Inflammatory Agents

##### Corticosteroids/Calcineurin Inhibitors

The use of topical corticosteroids (TCs) such as clobetasol or betamethasone in FFA remains controversial [17,45,46,49]. Some studies suggest that while TC use may result in stabilization in the early stages, discontinuation can be associated with relapse [17], and the treatment can also result in scalp skin atrophy, telangiectasia, erythema, or folliculitis [42,46]. In comparison, topical calcineurin inhibitors (TCIs) appear to have higher efficacy [47]. Patients receiving tacrolimus (0.3%) were more likely to achieve stabilization within 3 months compared to those treated with TCs [26]. However, the use of TCs or TCIs as monotherapy is ineffective and results in remission in 1–3% of cases [45], and they should hence be considered only as part of combined therapy.

##### JAK Inhibitors (JAKis)

Plante et al. report that combined therapy with 2% tofacitinib resulted in clinical improvement after 3 months [50]. Ruxolitinib treatment (1.5% twice daily), in combination with systemic agents, led to a significant reduction in erythema and pruritus after 12–15 weeks in two patients and hair regrowth in another patient [51]. In both studies, the main treatment modification was based on a change in topical medication, while the groups of systemic drugs remained unchanged. This suggests that achieving stabilization in FFA refractory to previous treatments may simply require an alteration in topical therapy, without the need for changes in systemic medications.

##### Mechlorethamine

Mechlorethamine induces lymphocyte apoptosis and reduces the number of Langerhans cells in the epidermis, these being the main population of antigen-presenting cells in the hair follicle. A pilot study evaluating the use of mechlorethamine (0.016% gel) involved topical application of the drug for 24 weeks in patients with FFA (n = 7) and LPP (n = 5). Partial and complete responses to treatment were achieved in 73% and 18% of patients, respectively; however, the efficacy was assessed using the LPPAI scale (the main tool for assessing LPP activity), the FFA patients were not analyzed as a separate group, and all patients developed contact dermatitis. This may be related to inflammatory activity in LPP/FFA, increasing the risk of primary antigen immunization and the development of acute contact dermatitis.

Due to the limited effectiveness of topical therapies for FFA, the use of mechlorethamine should be implemented only in patients with severe, treatment-resistant disease or as a short-term adjunct to topical treatment in patients receiving systemic therapy. Further research on its use is needed, with a particular emphasis on reducing acute contact dermatitis and improving its effectiveness in disease stabilization [52].

#### 6.1.2. Hair Growth Modulators

##### Bimatoprost

Bimatoprost is a synthetic analog of prostamide F2a, which induces the anagen phase in the hair follicle and leads to an increased number, thickness, and length of hair [53,54,55]. Bimatoprost (0.03% eye drops) applied twice daily to the eyebrow area leads to eyebrow regrowth within 6–9 months without causing adverse effects [54,55].

##### Minoxidil

Topical application of minoxidil (5% solution) was found to have a positive response in up to 72% of cases when used as part of combined therapy with intralesional triamcinolone acetonide (ILTA) injections or systemic agents [45,56]; as such, the drug may be an effective complement for combined treatment of FFA [56]. However, treatment can result in facial hypertrichosis, contact dermatitis, irritation, or exacerbation of seborrheic dermatitis [45].

#### 6.1.3. Phototherapy

##### Neodymium YAG Laser (Nd:YAG Laser)

One study examined the effect of Nd:YAG laser therapy in treating FFA. The laser was applied to the facial and frontotemporal area three times at monthly intervals. Four out of five patients reported improvement in at least one symptom: four experienced reduced pruritus, and two reported reduced pain and burning. Additionally, perifollicular hyperkeratosis and erythema was reduced, and hair loss was stabilized. No significant adverse effects were noted in patients [57].

##### Excimer Laser

Compared to other types of phototherapy, excimer laser treatment offers lower UV exposure, shorter treatment duration, and the ability to target specific areas of the skin without damaging adjacent healthy tissue [58,59,60]. Navalini et al. examined the effect of excimer laser treatment twice a week on 13 patients with FFA or LPP. A reduction in erythema, pain, or pruritus was noted after a series of 6–16 treatments, as well as hair regrowth. Two patients achieved remission [61]. It should be emphasized that in this study, FFA was classified as a type of LPP, and systemic therapy was not standardized. Further observations are necessary to assess the use of the excimer laser in FFA.

##### Low-Level Light Therapy (LLLT)

Photobiomodulation, known as low-level light therapy, involves the use of low-power light sources that do not emit ions [62,63,64]. LLLT is believed to act by increased adenosine triphosphate (ATP) synthesis, oxidative stress modification, and transcription factor induction. It is hypothesized that LLLT affects the hair growth cycle by preventing premature catagen and increasing the proliferation rate of active anagen-phase hair follicles [65,66,67]. In a study conducted on 16 women, eyebrow hair regrowth was observed after 10 sessions conducted once a week. Improvement was greater in patients with partial eyebrow loss. Patients did not report side effects during or after the treatment [68].

#### 6.1.4. Intralesional Treatment

##### Intralesional Triamcinolone Acetonide (ILTA) Injections

ILTA is an FDA-approved medication used for managing diverse inflammatory conditions, including skin disorders such as discoid lupus, lichen planus, or alopecia areata. This drug is consistently recommended in all hair loss treatment guidelines and for FFA, and ILTA is acknowledged as one of the most effective therapeutic options so far [17,19,45,47,56]. As part of combination therapy, ILTA results in a favorable outcome in up to 84% of cases [42]. Administration every 6–8 weeks can limit disease progression or lead to complete stabilization [46], while application every 3–6 months (at 20 mg/mL) can result in hair regrowth. This therapy is also effective for eyebrow loss, especially partial loss [17]. In daily practice, ILTA is used as a first-line treatment, mainly in combination with oral 5-alpha-reductase inhibitors (5-ARIs), hydroxychloroquine, retinoids, or tetracyclines [19,45,46]. Nevertheless, before consenting to ILTA, it is imperative to consider the potential risks, such as scalp stiffness, skin atrophy, and exacerbation of skin fibrosis in the advanced stages of FFA [45].

##### Platelet-Rich Plasma (PRP) and Plasma Rich in Growth Factors (PRGF)

PRP and PRGF contain growth factors that may positively influence hair growth and maintain the integrity of the hair follicle [69,70,71]. A retrospective study involving 118 patients showed that adding PRGF to the treatment regimen contributed to more frequent achievement of disease stabilization and reduction in inflammation, as well as hair regrowth [71]. In one reported case, the addition of PRP to systemic therapy for five treatments led to a reduction in erythema and perifollicular hyperkeratosis within 1 month [72].

Before implementing PRP or PRGF, it is important to consider that FFA could be associated with other autoimmune diseases, and it is recommended to check for the presence and specificity of antinuclear antibodies or autoimmune markers. Previous research suggests that FFA may be associated with SLE, polymyositis, RA, or autoimmune thyroid diseases.

### 6.2. Systemic Treatment

#### 6.2.1. 5-ARIs

These medications are often used as a first-line treatment in combination with topical therapy or ILTA [19,45,56]. Their use was found to elicit a favorable treatment response in 69% of cases [45]. In one study involving 355 patients with FFA, finasteride treatment achieved a positive response in 100% of cases: 53% (54/102) achieved stabilization, and 47% (48/102) showed improvement. In addition, dutasteride was found to be effective for disease stabilization (10/18) and clinical improvement (8/18) [4]. Finasteride treatment is typically based on an initial dose of 2.5 mg/day, with an increase to 5 mg/day in case of no improvement within 6 months, while dutasteride is typically used at a dose of 0.5 mg/week—0.5 mg/day [4,73,74]. Although 5-ARIs are generally considered safe [75], they are category X drugs in pregnancy and may cause male fetus feminization. Therefore, finasteride is recommended in women due to its shorter half-life compared to dutasteride. Effective contraception is also recommended [45,75]. Although 5-ARIs are considered the most effective drugs for FFA [4,17,45,56], further research is necessary to clarify their therapeutic mechanism. Many authors suggest that the positive treatment response may result from the high efficacy of the drugs against androgenetic alopecia, which often coexists with FFA [76].

#### 6.2.2. Retinoids

First-generation retinoids, such as isotretinoin (10–40 mg/day) and alitretinoin (30 mg/day), and second-generation ones, such as acitretin (20 mg/day), were effective in FFA [77,78,79,80]. In a study conducted by Rakowska et al., comparing the effectiveness of isotretinoin, acitretin, and finasteride, no disease progression was observed after 12 months of therapy in 76% of patients treated with isotretinoin, 73% of patients treated with acitretin, and 42% of patients treated with finasteride. Furthermore, the FFA did not progress for 24 months after therapy in 72% of patients treated with isotretinoin and 73% of patients treated with acitretin [80]. The use of oral vitamin A derivatives plays an important role in FFA cases presenting with FP. Isotretinoin added to the therapy at a dose of 10–40 mg/day reduces these lesions rapidly, in some cases within 2 weeks. Complete resolution usually occurs within 1–5 months. Retinoids are also effective in reducing pruritus, a common symptom in patients with FP [79,81].

#### 6.2.3. Hydroxychloroquine

Hydroxychloroquine is an antimalarial drug often used as a first-line therapy in FFA. Positive outcomes have been noted in 63% of patients [45]. The usual dose is 200–400 mg/day. The effectiveness of this drug increases with long-term use [82]. A positive response is observed after 6–12 months [45,83,84]. Moreover, hydroxychloroquine and 5-ARIs appear to demonstrate comparable efficacy, as confirmed by a randomized study involving 36 patients [85].

However, the possibility of side effects of chronic hydroxychloroquine use, such as retinopathy, gastrointestinal disorders, myopathy, and headaches, should be considered. Hydroxychloroquine is safe in pregnancy, unlike other oral medications such as 5-ARIs or retinoids [45,83,86].

#### 6.2.4. Tetracyclines

Tetracyclines are a group of antibiotics with documented anti-inflammatory properties. Studies indicate a positive response to therapy in 60% of cases. It is suggested that doxycycline at a dose of 100 mg/day may be effective in FFA patients with concurrent rosacea [19,45].

#### 6.2.5. JAKis

##### Tofacitinib

Tofacitinib, an inhibitor of JAK1, JAK3, and, to a lesser extent, JAK2, was the initial JAKi developed for treating autoimmune diseases. It was the first JAKi to be used in the systemic treatment of FFA. Treatment at a dose of 10 mg/day, in combination with other systemic drugs, resulted in disease stabilization and hair regrowth in two patients with FFA after 6–9 months [83]. Plante et al. [50] report a case where a lower dose, i.e., 5 mg/day for 4 months, also resulted in a positive outcome.

##### Baricitinib

Oral use of baricitinib (3.4 mg/day, 4 mg/day, 6.8 mg/day), a JAKi targeting JAK1 and JAK2, has also achieved promising results, especially in FFA unresponsive to previous treatment methods, such as topical and oral tofacitinib [51].

However, systemic JAKi therapy can result in weight gain, nausea, and increased risk of infections, and these should be taken into account before starting. It is also recommended to perform a complete blood count; assess kidney and liver function parameters and lipid levels; and screen for hepatitis B and C viruses, tuberculosis, and HIV [87,88].

#### 6.2.6. Oral Minoxidil

Oral monotherapy with minoxidil has shown efficacy in moderate eyebrow hair loss. A study of treatment in seven patients for 6 months (initial dose: 0.5–1.25 mg/day, increasing to 1.25–2.5 mg/day after 3 months) resulted in the complete regrowth of eyebrows in five patients and partial regrowth in two. No hypertrichosis was observed [89]. Oral minoxidil (1 mg/day) in combination with dutasteride (0.1 mg/day) also resulted in long-term disease stabilization [90].

#### 6.2.7. Naltrexone

Low doses of naltrexone (1–5 mg/day) may exhibit anti-inflammatory properties by antagonizing toll-like receptor 4 [91,92,93,94]. Regarding FFA treatment, naltrexone contributed to a reduction in erythema and trichodynia when used at a dose of 3 mg/day as an additional component of therapy. However, no reduction in pruritus or perifollicular hyperkeratosis was observed. The side effects of treatment include vivid dreams, insomnia, headaches, thirst, and xerostomia, and these should be taken into consideration before treatment [95].

#### 6.2.8. Pioglitazone

Pioglitazone is an agonist of the peroxisome proliferator-activated receptor gamma (PPAR-γ), used in the treatment of type 2 diabetes mellitus. Its effectiveness in FFA is controversial [96,97,98], and it is rarely used as part of combination therapy [45].

#### 6.2.9. Other Anti-Inflammatory and Immunosuppressive Agents

##### Oral Corticosteroids

Studies indicate a positive response to oral prednisone (0.5–1 mg/kg/day). However, the discontinuation of such therapy leads to disease relapse [49].

##### Calcineurin Inhibitors

Some authors suggest the use of cyclosporine (3–5 mg/kg/day) as a first-line treatment, although the results of most studies are controversial. Others propose the use of cyclosporine or mycophenolate mofetil in cases of non-response to previous therapies or recurrent FFA [19,45,99].

##### Methotrexate

To date, four cases of methotrexate monotherapy have been reported in FFA patients. Stabilization of the disease process was observed in only two of them [26,47,100,101,102].

### 6.3. Surgical Treatment

#### 6.3.1. Hair Transplantation (HT)

HT in FFA therapy remains controversial. Over half of patients experience a decrease in hair density or disease recurrence between 8 and 48 months post-surgery [103]. Assessing the effectiveness of this treatment is challenging due to the limited number of patients reported in the literature and differences in HT techniques. However, there is a consensus that HT in FFA patients should be considered only after disease stabilization lasting at least 1–2 years [56,104]. Furthermore, long-term patient observation post-transplantation is essential, with follow-up visits recommended every 4–6 months to detect early signs of FFA recurrence [56].

#### 6.3.2. Scalp Reduction Surgery

Scalp reduction surgery prior to HT resulted in a satisfactory outcome for one FFA patient. After 2 years of disease stability, the patient underwent scalp reduction surgery followed by HT. Six years later, the patient remained stable, without signs of hairline recession or loss of the transplanted follicular units [105].

#### 6.3.3. Eyebrow Transplantation

Eyebrow transplantation is a widely-accepted procedure resulting in positive long-term results in patients with eyebrow loss due to trauma or excessive plucking [106]. However, in FFA, the procedure remains controversial. Short-term and long-term outcomes are comparable with scalp HT for most patients. Hair follicles in the eyebrows are maintained during the first 12 months post-surgery but demonstrate progressive loss in subsequent years [107].

## 7. Summary

FFA treatment is challenging. Currently, the only FDA-approved drug for FFA treatment is triamcinolone acetonide, administered via intralesional injections. Clinical observations suggest that combined therapy is more effective than monotherapy [45]. Data from the literature suggest that ILTA, 5-ARIs, retinoids, and hydroxychloroquine are the most effective therapeutic options. First-line medications should be chosen individually, based on the drug safety profile, the FFA phenotype, and the presence of coexisting conditions [19,85].

As topical agents appear to be ineffective in monotherapy, they should be considered as a part of combined treatment. TCIs appear to have superior efficacy over TCs; therefore, the former should be prioritized as the primary option for topical therapy. Despite the promising potential of JAKis, their application has only been described in a few case series.

Intralesional therapies appear to be effective; however, treatment involves discomfort even with local anesthesia. In addition, they may not be as effective in the advanced stages of FFA due to potential exacerbation of scalp fibrosis.

Laser therapy and LLLT have demonstrated efficacy in clinical settings while maintaining a favorable side effect profile, and they thus merit particular attention. They may well represent a noteworthy adjunctive treatment option for patients with FFA.

When opting for systemic drugs, it is crucial to consider not only their efficacy but also the safety profile and the influence of any comorbidities associated with FFA. For example, 5-ARIs are recommended in the presence of concomitant androgenic alopecia; finasteride appears to be a better option than dutasteride within this drug class among female patients due to its shorter half-life. Moreover, patients with FP may benefit from oral retinoids, while patients with rosacea may benefit from tetracyclines. Low-dose naltrexone may be considered as an adjunctive therapy to reduce inflammatory symptoms.

While hair and eyebrow transplantation yields initial hair follicle maintenance, the treatment typically results in rejection. However, the effectiveness of scalp reduction surgery as an intervention to reduce therapeutic failure needs further investigation.

## 8. Further Perspectives

A crucial initial step towards approaching the frontal fibrosing alopecia treatment dilemma involves gaining a comprehensive understanding of the etiopathogenesis of FFA, which would facilitate identifying targets for potential drugs. This understanding would open the door to randomized clinical trials aimed at assessing the effectiveness and safety profiles of emerging treatment modalities.

Additionally, given the psychological distress that hair loss, which accompanies FFA, inflicts upon patients, investigations into potential hair regrowth and its correlation with disease severity, treatment modalities, and trichoscopy findings should be included. A positive response requires the early implementation of therapy, when the fibrosis has not yet affected all the follicular units; in this case, the stimulation and the reduced inflammation in the follicles may contribute to hair regrowth.

## Figures and Tables

**Table 1:** Diagnostic criteria proposed by Tolkachjov and Vañó-Galván [35,36]. Diagnosis requires two major criteria, or one major criterion and two minor criteria.

Major Criteria	Minor Criteria
Cicatricial alopecia of the frontal, temporal, or frontotemporal scalp and absence of follicular keratotic papules on the body. Diffuse bilateral eyebrow hair loss.	Perifollicular erythema, perifollicular hyperkeratosis, or solitary hair shafts on examination in the frontal/frontotemporal area. Histopathological features of cicatricial alopecia in the pattern of FFA/LPP on scalp biopsy. Involvement of additional FFA sites: occipital area, facial hair, sideburns, body hair. Facial papules. Preceding/concurrent symptoms, such as pain or pruritus at sites of involvement.

FFA—frontal fibrosing alopecia. LPP—lichen planopilaris.

**Table 2:** Five grades of FFA severity based on largest measure of cicatricial area [4].

Grade of Severity	Cicatricial Area Largest Measure [cm]
I	<1
II	1–2.99
III	3–4.99
IV	5–6.99
V	≥7

**Table 3:** FFASS—Frontal Fibrosing Alopecia Severity Score.

Signs and Symptoms	Grade	Punctuation
**Clinical symptoms**		
Hairline recession [cm]		
Frontal	>1 1–2.99 3–4.99 5–6.99 >=7	grade × 2
Temporal left		grade × 1
Temporal right		grade × 1
Eyebrow loss	No	0
	Partial	0.5
	Total	1
**Alopecia score**	the sum of clinical symptoms punctuation max. 21	
**Inflammation**		
Severity		
Perifollicular hyperkeratosis	No	0
	Mild	0.1
	Severe	0.2
Perifollicular erythema	No	0
	Mild	0.1
	Severe	0.2
Extent along the frontotemporal line [%]		
Perifollicular hyperkeratosis	No	0
	<25%	0.1
	>25%	0.2
Perifollicular erythema	No	0
	<75%	0.1
	>75%	0.2
**Associated symptoms**		
Pruritus		
Severity	No	0
	Mild	0.3
	Severe	0.6
Frequency	No	0
	Occasional	0.3
	Daily	0.6
Pain		
Severity	No	0
	Mild	0.3
	Severe	0.6
Frequency	No	0
	Occasional	0.3
	Daily	0.6
**Inflammation score**	the sum of inflammation and associated symptoms punctuation max. 4	
**FFASS**	the sum of alopecia score and inflammation score max. 25	

**Table 4:** Frontal Fibrosing Alopecia Global Staging Score [37].

Symptom	Grade	Punctuation
Scalp hair loss	None	0
	Minimal (<1 cm)	1
	Mild (1 to <3 cm)	2
	Moderate (3 to <5 cm)	3
	Severe (≥5 cm)	4
Eyebrow loss	None	0
	Partial	1
	Total loss in at least one eyebrow	2
Facial papules	None	0
	Some	1
Prominent forehead veins	None	0
	Some	1
Facial hyperpigmentation	None	0
	Some	1

**Table 5:** Selected treatment options for FFA. ILTA—intralesional triamcinolone acetonide. PRP/PRGF—Platelet-Rich Plasma/Plasma Rich in Growth Factors.

Local Therapy	Systemic Therapy	Surgical Procedures
**Topical agents**	**5-alpha-reductase inhibitors** Dutasteride Finasteride **Antimalarial agents** Hydroxychloroquine **Retinoids** Isotretinoin Acitretin Alitretinoin **Tetracyclines** Tetracycline Doxycycline **JAK inhibitors** Tofacitinib Baricitinib Minoxidil Pioglitazone Naltrexone **Calcineurin inhibitors** Cyclosporine Mycophenolate mofetil Azathioprine Oral corticosteroids	Hair transplantation Scalp reduction surgery Eyebrow transplantation
**Corticosteroids** Betamethasone Clobetasol **Calcineurin Inhibitors** Tacrolimus Pimecrolimus **JAK inhibitors** Tofacitinib Ruxolitinib Mechlorethamine **Hair Growth Modulators** Minoxidil Bimatoprost		
**Intralesional injections**		
ILTA * PRP/PRGF		
**Phototherapy**		
Low-Level Light Therapy Excimer Laser Nd:YAG		

* It is important to emphasize that the only drug registered by the Food and Drug Administration (FDA) for FFA treatment is triamcinolone acetonide, administered via intralesional injections, and, to date, no systemic drug has been approved.

## Data Availability

Data sharing is not applicable to this review as no new data were generated.
